# Editorial: Advances in thrombin generation

**DOI:** 10.3389/fcvm.2023.1183718

**Published:** 2023-03-30

**Authors:** Romy de Laat-Kremers, Stéphane Zuily, Bas de Laat

**Affiliations:** ^1^ Department of Data Analysis and Artificial Intelligence, Synapse Research Institute, Maastricht, Netherlands; ^2^ Vascular Medicine Division, CHU de Nancy, Nancy, France; ^3^ Department of Functional Coagulation, Synapse Research Institute, Maastricht, Netherlands

**Keywords:** thrombin generation, coagulation, hemostasis, coagulation assays, plasma, whole blood, thrombosis, bleeding

**Editorial on the Research Topic** Advances in thrombin generation

**Figure 1:**
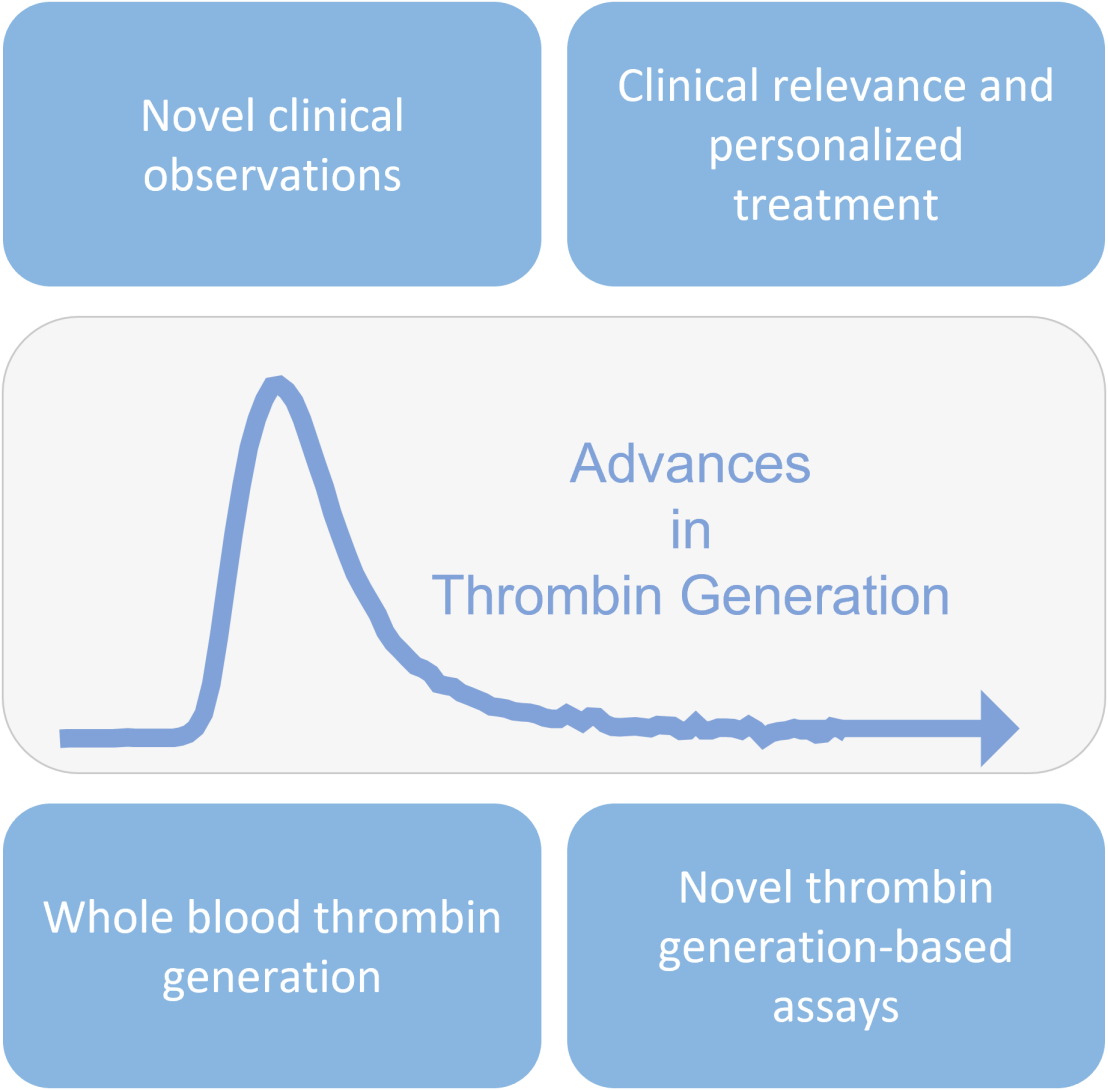
An overview of the topics in the articles in the advances in thrombin generation collection.

The thrombin generation (TG) test is a global coagulation assay (1). An increased TG has been associated with thrombosis (2–4), whereas a decreased TG can result in bleeding episodes (5–9). This collection of articles illustrates the recent clinical and technical advances that have been made in the field of thrombin generation. The articles in this collection describe novel clinical observations using the TG test, the clinical relevance of TG and its role in the personalization of treatment, the development and use of the TG assay in whole blood, and the development of new TG based assays (Figure 1).

Whereas TG used to be a typical research laboratory test, the development of fully automated TG analyzers has introduced TG to the clinical laboratory (10). As a result, TG has become of interest to support the personalization of treatment in bleeding and thrombotic diseases (Valke et al.). For example, TG-dependent activated protein C resistance is higher in antiphospholipid syndrome patients compared to controls (Gehlen et al.). A clear difference between patients and controls can be detected (11), although more research is needed to determine the diagnostic and prognostic value of TG in diagnostic strategies. Indeed, previous studies have shown that an imbalance of pro- and anticoagulant processes could be indicative of clinical outcomes in APS (12, 13). Therefore, the availability of the TG assay in the clinical laboratory is an important opportunity for TG-based personalized treatment. Carlo et al. describe the implementation of the thrombin dynamics method on ST Genesia acquired data. The thrombin dynamics method was originally developed for the semi-automated TG assay (Calibrated Automated Thrombinoscopy; CAT). Thrombin dynamics analysis allows a more in-depth analysis of TG curves measured on ST Genesia, bringing us one step closer to TG-based personalized medicine in antiphospholipid syndrome patients and other patient groups.

Clinical observational studies using the TG test have shown that TG remains high in COVID-19 patients that are admitted to the intensive care unit. Hence, increased TG is hypothesized to contribute to the prothrombotic phenotype in COVID-19 patients (van de Berg et al.). Moreover, vaccination against COVID-19 with ChAdOx1-S was associated with a prothrombotic TG profile with a shortened lag time and increased peak height in the weeks after vaccination (de Laat et al.). Furthermore, Feugray et al. show that TG can be used as an indicator for vaso-occlusive crisis in sickle cell disease patients. Additionally, TG analysis in the Moli-sani cohort (14) revealed that increased TG is associated with higher BMI and blood lipid levels with increased TG parameters (de Laat-Kremers et al.). These findings may partly explain the increased risk of cardiovascular diseases in individuals with obesity and/or dyslipidemia.

An important factor in the advancement of TG is the development of novel, specialized TG assay protocols, designed to detect either a specific patient phenotype or to shed more light on mechanistical defects in a patient population of interest. Bai et al. reports the development of specialized TG assays in which the individual role of FII, FV or FX in the TG process can be assessed in an individual. Calibrated Automated Thrombinoscopy (CAT) is the most widely used method for the measurement of TG worldwide (11). Under some circumstances, for example in pediatrics research, it is difficult to obtain the necessary volume of plasma samples to allow the measurement of TG (15). Nevertheless, the growing interest in TG has led to development of the MidiCAT, which uses only half the amount of sample required for the regular CAT (16). Charles et al. have externally validated the MidiCAT and report that the experimental variation for both the regular CAT and midi-CAT were low and that the agreement between MidiCAT and CAT to be satisfactory.

Another TG method that attracts more and more attention is the whole blood TG assay (17). Li et al. shows that multiple myeloma patients have a disbalanced whole blood TG profile, which may explain the paradoxically high prevalence of bleeding and increased risk of thrombo-embolism. Moreover, the whole blood TG assay was used to study the intrinsic coagulation pathway-mediated TG in mice (Konrath et al.), showing an additional advantage for whole blood TG in animal studies.

In conclusion, this collection gives an overview of the advances made in the field of thrombin generation, including but not limited to its usefulness in the clinic as a predictor for personalized therapeutic strategies, the assessment of hemostatic changes in COVID-19 patients and the validation of the MidiCAT for use in studies with small sample volumes. All together, the advances in the field of thrombin generation now allow the incorporation of the TG test in the clinical management of certain patient groups, and novel TG-based assays are expected to follow.
